# ESMSec: Prediction of Secreted Proteins in Human Body Fluids Using Protein Language Models and Attention

**DOI:** 10.3390/ijms25126371

**Published:** 2024-06-09

**Authors:** Yan Wang, Huiting Sun, Nan Sheng, Kai He, Wenjv Hou, Ziqi Zhao, Qixing Yang, Lan Huang

**Affiliations:** 1 Key Laboratory of Symbolic Computation and Knowledge Engineering of Ministry of Education, College of Computer Science and Technology, Jilin University, Changchun 130012, China; wy6868@jlu.edu.cn (Y.W.); htsun23@mails.jlu.edu.cn (H.S.); shengnan21@mails.jlu.edu.cn (N.S.); wjhou23@mails.jlu.edu.cn (W.H.); zqzhao21@mails.jlu.edu.cn (Z.Z.); yangqx22@mails.jlu.edu.cn (Q.Y.); 2 Department of Computational Medicine and Bioinformatics, University of Michigan, Ann Arbor, MI 48103, USA; kaihe@umich.edu

**Keywords:** disease biomarkers, protein language models, multi-head attention, human body fluid

## Abstract

The secreted proteins of human body fluid have the potential to be used as biomarkers for diseases. These biomarkers can be used for early diagnosis and risk prediction of diseases, so the study of secreted proteins of human body fluid has great application value. In recent years, the deep-learning-based transformer language model has transferred from the field of natural language processing (NLP) to the field of proteomics, leading to the development of protein language models (PLMs) for protein sequence representation. Here, we propose a deep learning framework called ESM Predict Secreted Proteins (ESMSec) to predict three types of proteins secreted in human body fluid. The ESMSec is based on the ESM2 model and attention architecture. Specifically, the protein sequence data are firstly put into the ESM2 model to extract the feature information from the last hidden layer, and all the input proteins are encoded into a fixed 1000 × 480 matrix. Secondly, multi-head attention with a fully connected neural network is employed as the classifier to perform binary classification according to whether they are secreted into each body fluid. Our experiment utilized three human body fluids that are important and ubiquitous markers. Experimental results show that ESMSec achieved average accuracy of 0.8486, 0.8358, and 0.8325 on the testing datasets for plasma, cerebrospinal fluid (CSF), and seminal fluid, which on average outperform the state-of-the-art (SOTA) methods. The outstanding performance results of ESMSec demonstrate that the ESM can improve the prediction performance of the model and has great potential to screen the secretion information of human body fluid proteins.

## 1. Introduction

The diverse array of proteins found within human body fluids serve as biomarkers for detecting and monitoring diseases, enhancing diagnostic accuracy, and assessing risk levels [1,2,3,4]. Because of this, the study of proteins secreted by human body fluids will become very necessary. The first identification of proteins in human body fluids dates back to 1937 [5]. Since then, with the development of proteomics technology, more proteins can be identified from human body fluids through techniques such as two-dimensional gel electrophoresis (2-DE) [6] and mass spectrometry (MS) [7]. For example, M.G. et al. identified a series of differentially expressed proteins associated with pancreatic cancer through pancreatic fluid analysis [8]. Similarly, D.C. et al. utilized MS methods to discover biomarkers in 1000 human blood samples [9]. However, high-precision mass spectrometry detection is often limited by expensive experimental costs. Therefore, fast and cost-effective bioinformatics-based research methods offer a new perspective for predicting body fluid protein profiles.

Machine-learning-based protein prediction methods have made significant strides in predicting various body fluids. Among these, the support vector machine (SVM) [10] prediction method stands out as a representative approach. This method employs binary classification to determine whether a protein is secreted into a specific human body fluid. The training process involves gathering a wide range of common protein features (sequence length, autocorrelation, hydrophobicity, charge, subcellular localization, longest disorder region, etc.) and then utilizing the recursive feature elimination (RFE) method based on SVM to select important protein features. Subsequently, the SVM model is employed to model proteins in body fluids. This approach has been successfully applied to studies involving saliva and urine [11,12]. While the feature-based model has shown promising results, it can be influenced by manual intervention during feature selection. In response to this limitation, neural network models leveraging deep learning (DL) techniques, such as convolutional neural networks (CNNs), fully connected neural networks, gated recurrent units (GRUs), and transformers, have been adopted to predict proteins in human bodily fluids. The advent of DL, fueled by increased data availability and high-capacity computer hardware, poses a challenge to traditional machine learning methods. One of the main advantages of DL lies in its ability to better represent raw data through nonlinear transformations, enabling more effective learning of hidden patterns within the data. Studies on transformer architecture [13] have demonstrated its efficacy in tackling large-scale computing challenges posed by excessively long sequences, surpassing CNNs in various tasks. For instance, Du et al. proposed a DL model for predicting secretory proteins in plasma and saliva [14]. Shao et al. learned complex features from protein sequence information through a CNN, a bidirectional gated recurrent unit (BGRU), and other networks, and completed the prediction of human body fluids. The model built was called DeepSec, which improved the prediction performance. However, the amount of protein data in body fluids is limited, so the model will be overfitted in many human fluids. Huang et al. extracted information from protein sequences through the densely connected convolutional networks (DenseNet) model and transformer architecture, etc. and proposed the DenSec model for predicting secreted proteins in cerebrospinal fluid (CSF) [15]. The prediction methods of DL use complex network structures, which result in a large number of parameters in the model. He et al. propose MultiSec, which predicts body fluids through multi-task learning, using less computational complexity to improve prediction accuracy [16]. The above studies are based on position-specific scoring matrix (PSSM) information to predict proteins, and it is necessary to propose a more efficient prediction method using other information to make the prediction more accurate.

In recent years, deep-learning-based language models (LMs) have achieved remarkable advancements in natural language processing (NLP). These deep learning LMs excel in tasks like predicting the next word in a sentence or reconstructing corrupted text to understand language based on contextual cues. Similarly, protein language models (PLMs) based on the transformer architecture have found success in the field of proteomics. PLMs are trained on extensive datasets of protein sequences to capture underlying evolutionary patterns and extract semantic information embedded within the protein sequences [17,18]. One of the basic pre-processing steps in NLP is tokenization, the splitting of the protein amino acid sequences into individual units of atomic information called tokens. Most NLP models use words as tokens, but some models use characters as tokens. Twenty basic amino acids make up human proteins, so the characters ‘A’, ‘C’, ‘D’, ‘E’, ‘F’, ‘G’, ‘H’, ‘I’, ‘K’, ‘L’, ‘M’, ‘N’, ‘P’, ‘Q’, ‘R’, ‘S’, ‘T’, ‘V’, ‘W’, and ‘Y’ are used to represent amino acids (‘A’ for alanine, etc.), which are modeled with a character-level PLM model. At present, the widely adopted PLMs include evolutionary scale modeling (ESM) [19] series models and ProtTrans series models. For instance, ESM-1b is a high-capacity transformer with protein sequence as input and hyperparameter optimization training. Post-training, the model’s output representation contains information about the structure, function, homology, and other secondary levels of the protein, and this information can be manifested by linear projection. The ProtTrans models have been developed to predict protein secondary structures for tasks like subcellular localization and membrane relative water solubility prediction. Notably, ProtT5 has achieved breakthroughs in secondary structure prediction, surpassing state-of-the-art methods without requiring multiple sequence alignment (MSA) or evolutionary information.

In this paper, we propose a model for predicting protein secretion in human body fluids, ESMSec. This model is composed of ESM2 (pre-trained esm2_t12_35M_UR50D, the embedding layer accepts a vocabulary of length 33, each word is embedded as a vector of length 480, and the fill tag index is 1 (<pad>)) [20] and attention architecture. Initially, the data are sampled in a balanced manner according to different body fluids, and the balanced protein amino acid sequence is input into the ESM2 model to extract the feature information of the sequence. Then, the extracted information is used as the input of multi-head attention architecture, and the output information is input to the feedforward neural network (FFN) and finally through the fully connected layer for binary classification. We selected plasma, CSF, and seminal fluid, which are three important and ubiquitous fluids, for the experiment. ESMSec achieved relatively accurate prediction in all human body fluids, with an average area under the receiver operating characteristic curve (AUC) of 0.9157, and it is proved that the ESM can extract protein secretion information.

## 2. Results

### 2.1. Performance of ESMSec in Three Human Body Fluids

In our study, ESMSec was developed using Python 3.10 and implemented primarily using PyTorch 1.12 and Scikit-Learn 1.2 [21,22]. The model training and testing were performed on a GeForce RTX 2080 Ti GPU. Comparison experiments were conducted on a Windows 11 platform. Firstly, to address the imbalance in positive and negative sample data across different human body fluids, a balanced sampling strategy was employed. This strategy generated three groups of data for each body fluid type, with a random selection ratio of 6:2:2 for training, validation, and testing datasets, respectively. Secondly, the pre-trained ESM2 model was utilized to extract features from the processed protein amino acid sequences, with sequence length controlled at 1000 and an output shape of 1000 × 480. Subsequently, a multi-head attention architecture and feedforward neural network (FFN) with a four-layer fully connected structure were used for protein sequence classification and prediction. The classification loss for each body fluid was calculated accordingly. The Adam optimizer was utilized to optimize the loss function for secreted proteins in each body fluid, with a learning rate set at 0.00005. ESMSec underwent 20 iterations with the training datasets, and the iteration with the highest accuracy (ACC) score for each body fluid was selected based on the corresponding validation datasets. After training, the ESMSec was evaluated on a testing dataset of three human body fluids, including plasma, CSF, and seminal fluid. Table 1 presents the benchmark test results for ESMSec on these testing datasets. ESMSec achieved performance ranging from 83.25% to 84.86% in ACC, 83.00% to 84.35% in F-measure (F1), 66.53% to 69.87% in Matthews correlation coefficient (MCC), and 90.73% to 92.76% in AUC. This indicated that ESMSec obtained good performance in the three body fluids simultaneously.

### 2.2. Evaluating the Performance of Classification

We conducted a performance comparison of ESMSec with various existing methods, including SVM-based, decision tree (DT)-based, DNN-based, DeepSec-based, MultiSec-based, and ESM-1b-based [19] methods. The hyperparameters for these methods were chosen based on the MCC metric from the validation dataset, and their performance on the testing dataset is reported as the benchmark for comparison.

- SVM is established based on protein features because SVM cannot directly model protein sequences, Initially, computational tools (UniProt, Profea, etc.) are employed to calculate features based on protein amino acid sequences, and the SVM-RFE method is applied for the iterative selection of collected features. The top 50 significant features are then chosen using the T-test and false discovery rate (FDR), and the SVM classifier is used to predict protein secretion in specific body fluids. The maximum number of iterations is 300, and the default values are used for other parameters;
- The modeling process of the DT-based method is similar to the SVM method. The depth of the DT model is 7, and the minimum number of samples required to split the internal nodes is 20;
- In the DNN model, the input feature dimension is 50, the number of neurons is 500, the number of layers is 4, the learning rate is 0.0001, and the batch size is 32;
- DeepSec bypasses feature collection and selection, opting for end-to-end training via protein PSSM data. It addresses the imbalance issue through a bagging strategy, training multiple networks simultaneously to identify secreted proteins within a single body fluid, which demands significant computational time and resources. Fifty filters of different sizes of {1, 5, 7} were utilized to extract features and combined to obtain a 1000 × 150 feature map with a learning rate of 0.0001;
- MultiSec adopts a balanced sampling strategy to solve the imbalance problem, trains the network through the multiple gradient descent algorithm (MGDA), builds a lightweight CNN to extract feature information, and uses a multi-task method to predict protein secretion. It extracts protein features at different scales via multiple parallel convolution layers, incorporating four parallel convolution and pooling operations. The filter sizes are {3, 5, 7, 9}, with 128 filters and a learning rate of 0.0001.

**Figure 1:**
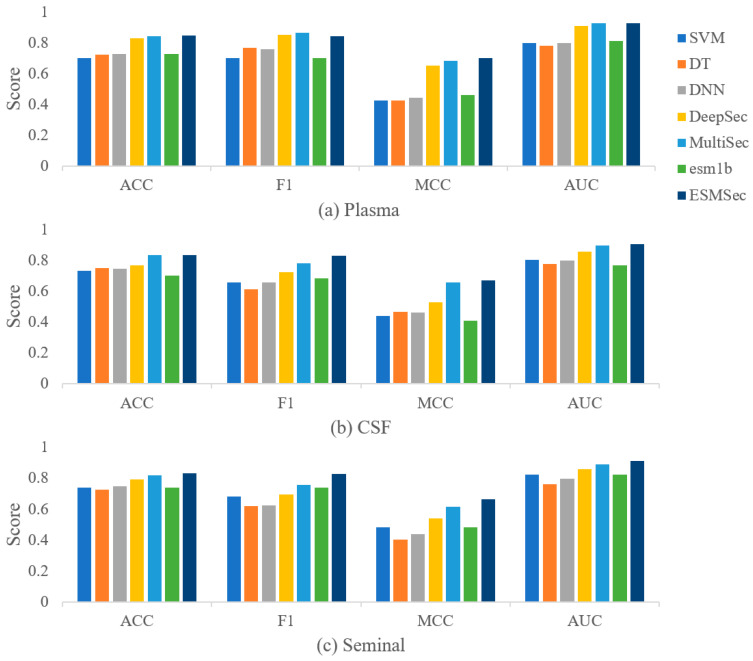
Comparative baseline methods for test datasets corresponding to 3 human body fluids. (**a**) In the plasma testing dataset; (**b**) in the CSF testing dataset; (**c**) in the seminal fluid testing dataset. (ACC: Accuracy, F1: F-measure, MCC: Matthews correlation coefficient, AUC: Area under curve).

For our method, the dropout in our FFN is set to 0.3 in plasma and seminal fluid and 0.2 in CSF. We employ the same model architecture to train three models. To ensure experimental fairness, we also compare with the pre-trained ESM-1b model, which shares the same structure as ESMSec. Table 2 presents the average benchmarks for ESMSec and other methods. As depicted in the table, our classifier outperforms other methods on average in ACC, F1, MCC, and AUC. (The methodological evaluation index scores of the three body fluids are shown in Table A1, Table A2, Table A3 and Table A4 of Appendix A). Figure 1 illustrates the average performance of the three body fluids across the seven classifiers, with our method achieving the highest overall average score. Considering various evaluation metrics, ESMSec demonstrates superior accuracy in predicting the likelihood of identifying secreted proteins compared to other methods, further confirming the ESM’s efficacy in extracting distinctive protein characteristics.

**Figure 2:**
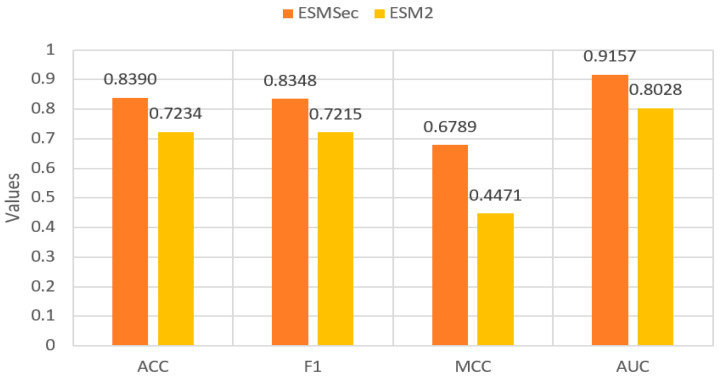
Results of the ablation experiment.

To assess the effectiveness of our proposed ESMSec approach, we conducted ablation experiments, and the results are shown in Figure 2, providing a comprehensive insight into our method’s performance. The figure clearly shows that our method outperforms the ESM2 method on average for the three body fluid testing datasets. This finding underscores the advantage of incorporating attention architecture in protein classification.

### 2.3. Prediction of Potential Secreted Proteins

ESMSec was utilized to identify potential secreted proteins in three types of human body fluids. We collected 8691, 9714, and 9049 proteins from plasma, CSF, and seminal fluid, respectively, which were not experimentally verified. We retrained the ESMSec, and for the prediction of the protein, we predicted the proteins with a probability greater than 0.5 as the potential proteins in the corresponding human body fluid, in which the predicted number of proteins in plasma is 5919 (As shown in Supplementary Materials Table S1), in CSF the predicted number of proteins is 6728 (As shown in Supplementary Materials Table S2), and in seminal fluid the predicted number of proteins is 5885 (As shown in Supplementary Materials Table S3). Table 3 shows the information of the five proteins with the highest prediction probability for each body fluid. In addition, through consulting relevant literature, a total of seven of the most important proteins in the three body fluids predicted by us have been verified as corresponding body fluid proteins by experiments.

## 3. Discussion

ESMSec is a computational model that leverages PLM to predict secreted proteins across various human body fluids. It utilizes the ESM to extract embedded features, which are then processed through a multi-head attention mechanism and a fully connected neural network. Compared to methods based solely on protein features and PSSM, ESMSec demonstrates higher prediction accuracy and superior generalization performance. This highlights the capability of the ESM in extracting information related to secreted proteins in human body fluids. On average the F1 metrics for the three human fluids show that our method outperforms the best-performing method (MultiSec) from other approaches by about 3.39% on the testing dataset. This indicates that ESMSec effectively represents proteins across the protein space. By incorporating the attention framework, our model can better capture long-distance dependencies, leading to the identification of 5919, 6728, and 5885 potential secreted proteins in the three body fluids. These findings open up new possibilities for future biological experiments.

By comparing models with different parameters in the ESM2 series, we finally selected a 12-layer model with a parameter count of 35M, which outperformed the other parameter count models on average across all body fluids. Due to limited hardware resources, only four ESM2 models could be used for experiments (ESM2_t33_650M runs on GeForce RTX 3090 GPU). The average evaluation indexes of the three body fluid testing datasets are shown in Table 4 (The index scores of the three body fluids on ESM2 models of different sizes are shown in Table A5, Table A6 and Table A7 of Appendix A).

However, it is evident from all the experimental methods that the MCC index is generally low, while the AUC index remains high. This analysis suggests that the imbalance in the classification threshold may be the cause, as the MCC value can fluctuate with changes in this threshold. Taking all this information into account, we have full confidence in the predictive capabilities of our method. Although ESMSec has achieved good prediction results, there is still room for optimization. In the future, we will improve the performance of prediction accuracy through input methods such as simultaneous input and collect more data to test different body fluids. We also need to investigate further the specificity of the protein in different body fluids and work to improve the interpretability of its entry into body fluids to make this approach more meaningful.

## 4. Materials and Methods

### 4.1. Data Collection

The data utilized in this study were sourced from the Human Body Fluid Proteome (HBFP) open database, which collected 15,480 experimentally verified proteins in body fluids from 241 articles. We specifically focused on plasma, CSF, and seminal fluid from this database for our experiments and searched proteins secreted by the three types of human body fluids and corresponding sequences from the database. Based on these data, three sub-datasets were constructed respectively. For each data subset, the positive sample was the experimentally verified in body fluid protein in the database, and the negative sample was generated by the positive sample data and the Pfam protein family information [23]. Specifically, first, all human proteins are obtained from the UniProt database and mapped to the corresponding Pfam family, then all the Pfam family information is found in the positive sample dataset, all the proteins in the Pfam family are removed, and finally, for each family, if the protein belongs to the family and the family intersects with the secreted protein, it is not taken as a negative sample, and if the protein does not belong to any family that meets the conditions, it is taken as a negative sample of the current body fluid. To ensure an accurate evaluation of our protein prediction method, we filtered out redundant proteins using a sequence similarity approach. Initially, we calculated the sequence similarity of all proteins in the dataset using the PSI-CD-HIT program. Subsequently, one protein with over 90% sequence similarity was randomly retained, and the remaining proteins were removed as redundant [24]. The number of positive and negative samples for each body fluid is shown in Table 5.

Considering the varying numbers of positive and negative samples, we applied balanced sampling to even out the data distribution. Each sub-dataset was then randomly divided into training, validation, and test datasets in a 60%, 20%, and 20% ratio, respectively. The training dataset was utilized for method training, the validation dataset for parameter selection, and the testing dataset for evaluating prediction performance. The distribution data of proteins in human body fluids are shown in Table 6, and the range of sequence lengths in each body fluid is shown in Table 7.

### 4.2. Model

**Figure 3:**
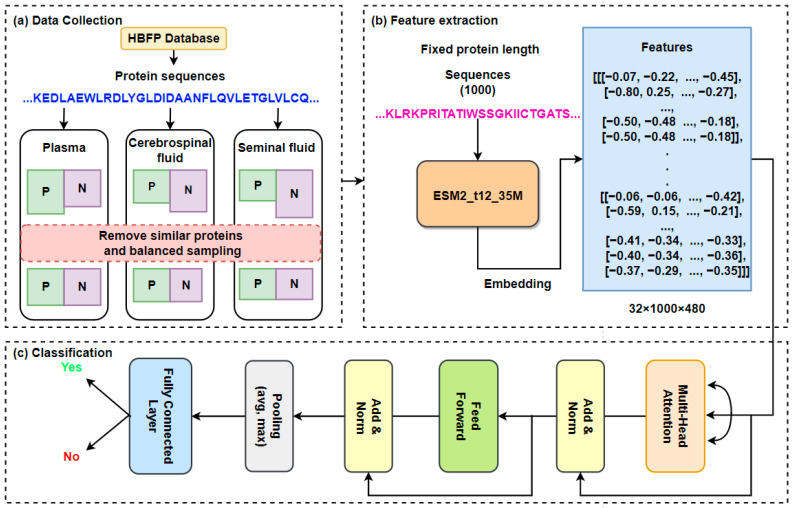
ESMSec architecture diagram ((**a**) Data Collection. (**b**) Feature extraction. (**c**) Classification).

In this paper, ESMs and attention architecture were used to predict secreted proteins in plasma, CSF, and seminal fluids. The overall architecture is shown in Figure 3. First, the input to the model is protein sequence information, rather than electing for the traditional PSSM, and then the features of the protein sequence are captured through the ESM2 model. Finally, the multi-head attention architecture with full connection and FFN is utilized as the classifier of whether the protein enters the corresponding body fluid.

#### 4.2.1. Feature Extraction

Since the ESM has been utilized for feature extraction of protein amino acid sequences, this model was also used for feature extraction of the sequence of protein data in body fluids in this study. The collected protein amino acid sequences undergo a pre-processing step where sequences are standardized to a fixed length. If a protein sequence exceeds 1000 residues, we concatenate the first 500 residues with the last 500 residues to ensure uniformity. Subsequently, we tokenize the sequence information using the ESM. (We chose the data of length 1000 for the experiment. Long sequences of proteins lose a lot of information, but in our data, about 12% of the data are affected by truncation, so if there is missing information, the negative impact on our method will not be very large). Finally, we extract the embedded information from the last layer of the protein language model (PLM) to obtain a dimensional representation of 1000 × 480.

#### 4.2.2. Classification

The classification module can calculate the probability that the protein will be secreted into a certain body fluid based on the features extracted by the final ESM module. A batch size of 32 was utilized, resulting in a dimension of 32 × 1000 × 480. Subsequently, the relationships within the sequence are captured by a multi-head attention mechanism, and then feature extraction and cross-layer information transfer are carried out by a fully connected feedforward network with residual connection, and layer normalization is used to stabilize the training process of the model.

(1) $$Attention(X,X,X)=soft\max(\frac{XX^{T}}{\sqrt{d_{x}}})X$$

where X is the embedded feature of the ESM2 output, repeated three times as the query, key, and value, the scaling factor is $\frac{1}{\sqrt{d_{x}}}$. The result is output after being calculated by the attention mechanism.

(2) $$MultiHead(X,X,X)=Concat(head_{1},\ldots ,head_{8})W^{O}$$

(3) x=LN(X+MultiHead(X,X,X))

(4) $$FFN(x)=\max(0,xW_{1}+b_{1})W_{2}+b_{2}$$

(5) h=LN(x+FFN(x))

(6) $$y_{kij}=\underset{(p,q)\in {ℜ}_{ij}}{\max}h_{kpq}$$

(7) $$p_{kij}=\frac{1}{\left|{ℜ}_{ij}\right|}\sum_{(p,q)\in {ℜ}_{ij}}h_{kpq}$$

(8) $$q=Concat(y_{kij},p_{kij})$$

The MultiHead is a multi-head attention operation, the LN layer is a normalized operation, and FFN is a feedforward neural network, which consists of two linear transformations. The first layer will change the dimension by four times first and add the GELU function in the middle. W and b are the weight vector and bias, respectively, and h is the result of the second LN layer. In the pooling layer, maximum pooling and average pooling concat are used to obtain two dimensions of the initial dimension which is put into the final fully connected layer (q).

(9) f=max(0,q⋅μ+ν)

This method is a fully connected layer composed of four hidden layers and carries out nonlinear transformation, where μ and ν are the weight vector and the bias. For prediction, we use softmax as the activation function at the output layer, and then cross-entropy loss as the loss function for binary classification, which is defined below:

(10) $$L=\frac{1}{n}\sum_{i=1}^{n}-(y_{i}\cdot \log({\hat{y}}_{i})+(1-y)\cdot \log(1-{\hat{y}}_{i}))$$

where $\hat{y}$ and y, respectively, represent the predicted value and the true value, n is the number of proteins. When predicting proteins in body fluids, the category corresponding to the larger output is selected as the prediction label.

#### 4.2.3. Evaluation

In the experimental comparison, we selected four evaluation indicators of ACC, F1, MCC, and AUC. It is worth noting that higher values indicate better classification performance for all those measures. These metrics are defined as follows:

(11) $$ACC=\frac{TP+TN}{TP+FP+FN+FN},$$

(12) $$F1=\frac{2TP}{2TP+FP+FN},$$

(13) $$MCC=\frac{TP\times TN-FN\times FP}{\sqrt{\left(TP+FN)(TP+FP)(TN+FP)(TN+FN\right)}},$$

where TP TN, FP, and FN represent the number of protein samples corresponding to true positive, true negative, false positive, and false negative, respectively.

## 5. Conclusions

In this work, we present the novel method ESMSec for predicting secreted proteins in plasma, CSF, and seminal fluid, which consists of an ESM2 with 12 layers and 35M parameters and attention architecture. The embedded PLMs extracted the protein amino acid sequence information in body fluids without using standard feature extraction methods such as MSA. The method is evaluated using an HBFP database dataset, and the experimental results show that our method has a better predictive effect than other existing methods in terms of average evaluation indicators. In addition, we also introduced the processing methods of positive and negative data samples and compared SVM, DT, DNN, DeepSec, MultiSec, and ESM-1b, as well as carried out an ablation experiment using only the ESM2 model. The ACC of our method reached 83.90%, and the results of F1, MCC, and AUC are better than those of other methods. In the Discussion section, we also explained why we chose the ESM2 model with 12 layers and 35M parameters. Features extracted by PLMs have more information content than those extracted by other feature extraction methods in the existing research. From the data point of view, our method still has shortcomings because the use of PLMs requires more training data, and some data that are less related in other body fluids cannot achieve good results. We will continue to collect more data and test more data on other proteins entering body fluids to improve the accuracy of predicting proteins entering body fluids.

## Figures and Tables

**Table 1:** ESMSec benchmarking on independent testing datasets of 3 human body fluids.

Fluid Name	ACC	F1	MCC	AUC
Plasma	0.8486	0.8435	0.6987	0.9276
CSF	0.8358	0.8310	0.6726	0.9073
Seminal	0.8325	0.8300	0.6653	0.9123
Average	0.8390	0.8348	0.6789	0.9157

**Table 2:** Average benchmarks for ESMSec and other methods were compared on 3 independent testing datasets of human body fluids.

Method	ACC	F1	MCC	AUC
DT	0.7327	0.6665	0.4329	0.7737
SVM	0.7244	0.6802	0.4493	0.8090
DNN	0.7412	0.6808	0.4486	0.7978
DeepSec	0.7971	0.7583	0.5743	0.8744
MultiSec	0.8333	0.8009	0.6507	0.9056
ESM-1b	0.7235	0.7080	0.4505	0.8008
ESMSec	**0.8390**	**0.8348**	**0.6789**	**0.9157**

The best results are in bold.

**Table 3:** 5 Protein information with the highest prediction probability in 3 body fluids.

Fluid Name	Accession	Prediction Probability	Evidence
Plasma	Q96MM7	0.99043	Unconfirmed
Plasma	P33897	0.98953	PMID: 27067449
Plasma	P40126	0.98423	PMID: 33200256
Plasma	Q96NM4	0.97802	Unconfirmed
Plasma	Q969N4	0.96579	Unconfirmed
CSF	Q16820	0.99735	PMID: 34359689
CSF	P51784	0.98942	Unconfirmed
CSF	Q16600	0.98172	PMID: 34867169
CSF	Q12891	0.98143	PMID: 26515055
CSF	Q9UKS6	0.97689	Unconfirmed
Seminal	Q8WU67	0.99195	Unconfirmed
Seminal	Q9Y6X5	0.98892	PMID: 35930312
Seminal	P30486	0.97895	PMID: 31002754
Seminal	O95678	0.97253	Unconfirmed
Seminal	O94933	0.96922	Unconfirmed

**Table 4:** The evaluation indexes of ESM2 series models were compared on 3 body fluid testing datasets.

ESM2 Model	ACC	F1	MCC	AUC
ESM2_t6_8M	0.8100	0.8067	0.6225	0.8947
ESM2_t12_35M	**0.8390**	**0.8348**	**0.6789**	**0.9157**
ESM2_t30_150M	0.8050	0.7995	0.6115	0.8830
ESM2_t33_650M	0.7828	0.7820	0.5659	0.8587

The best results are in bold.

**Table 5:** The number of samples of 3 human body fluids.

Fluid Name	Positive	Negative
Plasma	6530	4856
CSF	4082	6281
Seminal	3929	7230

**Table 6:** Partitioning data of proteins in 3 human body fluids.

Fluid Name	Training Dataset	Validation Dataset	Testing Dataset
Plasma	5828	1942	1942
CSF	4900	1632	1632
Seminal	4716	1572	1570

**Table 7:** Sequence length range of 3 human body fluids.

Sequence Length Range	Fluid Name			Proportion
	Plasma	CSF	Seminal	
<500	6025	5049	5133	62.98%
500–1000	2374	2100	1925	24.87%
>1000	1313	1015	800	12.16%

## Data Availability

Data and code that support the reported results can be found at https://github.com/BBT-123/ESMSec (accessed on 20 April 2024).

## Supplementary Materials

The following supporting information can be downloaded at: https://github.com/BBT-123/ESMSec (accessed on 20 April 2024).

## Appendix A

Here, we give all the benchmarks for comparison experiments. Table A1, Table A2, Table A3 and Table A4 show the benchmarks for ACC, F1, MCC, and AUC indicators for all methods. Table A5, Table A6 and Table A7 show the benchmarks of ACC, F1, MCC, and AUC for each body fluid of the ESM2 series model.

**Table A1:** On the independent testing datasets, 7 methods of 3 kinds of human body fluid were compared on the ACC evaluation index.

Fluid Name	SVM	DT	DNN	DeepSec	MultiSec	ESM-1b	ESMSec
Plasma	0.6992	0.7224	0.7268	0.8300	0.8445	0.7271	**0.8486**
CSF	0.7321	0.7514	0.7486	0.7683	**0.8378**	0.7034	0.8358
Seminal	0.7418	0.7243	0.7481	0.7929	0.8176	0.7401	**0.8325**

The best results are in bold.

**Table A2:** On the independent testing datasets, 7 methods of 3 kinds of human body fluid were compared on the F1 evaluation index.

Fluid Name	SVM	DT	DNN	DeepSec	MultiSec	ESM-1b	ESMSec
Plasma	0.7002	0.7663	0.7584	0.8523	**0.8642**	0.7012	0.8435
CSF	0.6597	0.6148	0.6588	0.7254	0.7829	0.6837	**0.8310**
Seminal	0.6807	0.6183	0.6253	0.6972	0.7556	0.7391	**0.8300**

The best results are in bold.

**Table A3:** On the independent testing datasets, 7 methods of 3 kinds of human body fluid were compared on the MCC evaluation index.

Fluid Name	SVM	DT	DNN	DeepSec	MultiSec	ESM-1b	ESMSec
Plasma	0.4278	0.4271	0.4446	0.6522	0.6824	0.4611	**0.6987**
CSF	0.4389	0.4686	0.4640	0.530.	0.6565	0.4101	**0.6726**
Seminal	0.4813	0.4031	0.4373	0.5406	0.6133	0.4803	**0.6653**

The best results are in bold.

**Table A4:** On the independent testing datasets, 7 methods of 3 kinds of human body fluid were compared on the AUC evaluation index.

Fluid Name	SVM	DT	DNN	DeepSec	MultiSec	ESM-1b	ESMSec
Plasma	07969	0.7823	0.7980	0.9085	0.9266	0.8127	**0.9276**
CSF	0.8056	0.7789	0.7991	0.8571	0.8998	0.7669	**0.9073**
Seminal	0.8242	0.7600	0.7963	0.8576	0.8904	0.8228	**0.9123**

The best results are in bold.

**Table A5:** Index scores of 3 body fluid testing datasets on ESM2_t6_8M model.

ESM2_t6_8M	ACC	F1	MCC	AUC
Plasma	0.8229	0.8304	0.6483	0.9097
CSF	0.8070	0.7943	0.6187	0.8892
Seminal	0.8000	0.7953	0.6006	0.8853

**Table A6:** Index scores of 3 body fluid testing datasets on ESM2_t30_150M model.

ESM2_t30_150M	ACC	F1	MCC	AUC
Plasma	0.8141	0.8142	0.6282	0.8947
CSF	0.7972	0.7858	0.5978	0.8730
Seminal	0.8038	0.7984	0.6085	0.8814

**Table A7:** Index scores of 3 body fluid testing datasets on ESM2_t33_650M model.

ESM2_t30_150M	ACC	F1	MCC	AUC
Plasma	0.8048	0.8013	0.6101	0.8790
CSF	0.7665	0.7641	0.5332	0.8348
Seminal	0.7771	0.7807	0.5544	0.8622

## References

[1] Aronson J.K., Ferner R.E. (2017). Biomarkers—A general review. Curr. Protoc. Pharmacol..

[2] Hu S., Loo J.A., Wong D.T. (2006). Human body fluid proteome analysis. Proteomics.

[3] Huang L., Shao D., Wang Y., Cui X., Li Y., Chen Q., Cui J. (2021). Human body-fluid proteome: Quantitative profiling and computational prediction. Brief. Bioinform..

[4] Lathrop J.T., Anderson N.L., Anderson N.G., Hammond D.J. (2003). Therapeutic potential of the plasma proteome. Curr. Opin. Mol. Ther..

[5] Tiselius A. (1937). Electrophoresis of serum globulin: Electrophoretic analysis of normal and immune sera. Biochem. J..

[6] Margolis J., Kenrick K. (1969). Two-dimensional resolution of plasma proteins by combination of polyacrylamide disc and gradient gel electrophoresis. Nature.

[7] Zhao Y.-Y., Lin R.-C. (2014). UPLC–MSE application in disease biomarker discovery: The discoveries in proteomics to metabolomics. Chem. Biol. Interact..

[8] Grønborg M., Kristiansen T.Z., Iwahori A., Chang R., Reddy R., Sato N., Molina H., Jensen O.N., Hruban R.H., Goggins M.G. (2006). Biomarker discovery from pancreatic cancer secretome using a differential proteomic approach* S. Mol. Cell. Proteom..

[9] Cominetti O., Núñez Galindo A., Corthésy J., Oller Moreno S., Irincheeva I., Valsesia A., Astrup A., Saris W.H., Hager J., Kussmann M. (2016). Proteomic biomarker discovery in 1000 human plasma samples with mass spectrometry. J. Proteome Res..

[10] Cui J., Liu Q., Puett D., Xu Y. (2008). Computational prediction of human proteins that can be secreted into the bloodstream. Bioinformatics.

[11] Sun Y., Du W., Zhou C., Zhou Y., Cao Z., Tian Y., Wang Y. (2015). A computational method for prediction of saliva-secretory proteins and its application to identification of head and neck cancer biomarkers for salivary diagnosis. IEEE Trans. Nanobiosci..

[12] Wang Y., Du W., Liang Y. PUEPro: A computational pipeline for prediction of urine excretory proteins. Advanced Data Mining and Applications (ADMA). Proceedings of the Advanced Data Mining and Applications: 12th International Conference, ADMA 2016.

[13] Bahdanau D., Cho K., Bengio Y. (2014). Neural machine translation by jointly learning to align and translate. arXiv.

[14] Du W., Zhao X., Sun Y., Zheng L., Li Y., Zhang Y. (2021). SecProCT: In silico prediction of human secretory proteins based on capsule network and transformer. Int. J. Mol. Sci..

[15] Huang L., Qu Y., He K., Wang Y., Shao D. (2022). DenSec: Secreted Protein Prediction in Cerebrospinal Fluid Based on DenseNet and Transformer. Mathematics.

[16] He K., Wang Y., Xie X., Shao D. (2022). MultiSec: Multi-Task Deep Learning Improves Secreted Protein Discovery in Human Body Fluids. Mathematics.

[17] Alkuhlani A., Gad W., Roushdy M., Voskoglou M.G., Salem A.-b.M. (2022). PTG-PLM: Predicting Post-Translational Glycosylation and Glycation Sites Using Protein Language Models and Deep Learning. Axioms.

[18] Marquet C., Heinzinger M., Olenyi T., Dallago C., Erckert K., Bernhofer M., Nechaev D., Rost B. (2022). Embeddings from protein language models predict conservation and variant effects. Hum. Genet..

[19] Rives A., Meier J., Sercu T., Goyal S., Lin Z., Liu J., Guo D., Ott M., Zitnick C.L., Ma J. (2021). Biological structure and function emerge from scaling unsupervised learning to 250 million protein sequences. Proc. Natl. Acad. Sci. USA.

[20] Lin Z., Akin H., Rao R., Hie B., Zhu Z., Lu W., Smetanin N., Verkuil R., Kabeli O., Shmueli Y. (2023). Evolutionary-scale prediction of atomic-level protein structure with a language model. Science.

[21] Paszke A., Gross S., Massa F., Lerer A., Bradbury J., Chanan G., Killeen T., Lin Z., Gimelshein N., Antiga L. (2019). Pytorch: An imperative style, high-performance deep learning library. Adv. Neural Inf. Process. Syst..

[22] Pedregosa F., Varoquaux G., Gramfort A., Michel V., Thirion B., Grisel O., Blondel M., Prettenhofer P., Weiss R., Dubourg V. (2011). Scikit-learn: Machine learning in Python. J. Mach. Learn. Res..

[23] El-Gebali S., Mistry J., Bateman A., Eddy S.R., Luciani A., Potter S.C., Qureshi M., Richardson L.J., Salazar G.A., Smart A. (2019). The Pfam protein families database in 2019. Nucleic Acids Res..

[24] Huang Y., Niu B., Gao Y., Fu L., Li W. (2010). CD-HIT Suite: A web server for clustering and comparing biological sequences. Bioinformatics.

